# Complete remission and long-term survival of a patient with melanoma metastases treated with high-dose fever-inducing Viscum album extract: A case report

**DOI:** 10.1097/MD.0000000000008731

**Published:** 2017-11-17

**Authors:** Paul G. Werthmann, Alexander Hintze, Gunver S. Kienle

**Affiliations:** a Institute for Applied Epistemology and Medical Methodology (IFAEMM) at the University of Witten/Herdecke, Freiburg i. Brsg., Germany; b Klinik Arlesheim, Arlesheim, Switzerland; c Center for Complementary Medicine, Institute for Environmental Health Sciences and Hospital Infection Control, Medical Center – University of Freiburg, Freiburg, Germany.

**Keywords:** complete remission, fever, malignant cutaneous melanoma, Mistletoe, Viscum album extract

## Abstract

**Introduction:**

Metastatic malignant cutaneous melanoma (MCM)—a highly immunogenic cancer—typically has a poor prognosis. Viscum album extracts (VAEs) have strong immune-stimulating, apoptogenic, and cytotoxic effects.

**Case presentation:**

A 66-year-old MCM patient with newly diagnosed lymph node metastases opted for sole VAE treatment. VAEs were initially applied subcutaneously, and then later in exceptionally high, fever-inducing doses, both intravenously and intralesionally. The metastases shrunk over the following months, and after 2 years, all lesions had completely remitted (regional and hilar lymph nodes). The patient has been tumor free for 3.5 years at the time of publication (and for 5 years since initiation of intensified VAE treatment). Besides fever and flu-like symptoms, no side effects occurred.

**Discussion:**

We presume that VAE triggered an increased release of tumor-associated antigens, enhanced immunologic recognition, and increased immune response against the tumor tissue and induced tumor remission.

## 1 Introduction

Malignant cutaneous melanoma (MCM) is the sixth most common of all cancers and the deadliest of all skin cancers, with increasing incidence worldwide (incidence and mortality are 9.3–10.2 and 1.2–2.0 per 100,000 people, respectively). MCM has a fair prognosis in local disease (20-year survival of 75%) but a dismal prognosis in metastatic disease (5-year survival of 5–19%). Risk factors are male gender, light skin (non-Hispanic whites), increased ultraviolet (UV) index (intense, intermittent sun exposure; tanning beds), and immunosuppression. Complete surgical excision is the standard of care for early melanoma treatment and can cure minimally invasive and in situ lesions. Excision of lymph nodes and systemic adjuvant immunotherapy is recommended for locally advanced disease. Metastatic disease is treated with surgical metastasectomy, immunotherapy, targeted therapies, and radiation therapy. Chemotherapy is restricted to patients whose disease cannot be controlled with immunotherapy or targeted agents.^[1]^

MCM shows a high immunogenicity and has played a key role in the development of tumor immunology. Of particular interest is the unusually high rate of spontaneous regression of primary lesions (3.7–15% but without impacting survival); a fair rate of spontaneous remission of metastatic disease (about 0.23% with a positive impact on survival); regression of MCM after infections or immunologic active treatments; and lymphocyte infiltration in regressed lesions and depigmentation of the site of regression.^[2]^ Furthermore, an increased number of infections and episodes of high fever, as well as previous history of certain vaccinations, are correlated with a reduced risk of developing MCM.^[3,4]^ These findings have driven research into the complex and dynamic interactions between the immune system and melanoma cells, resulting in the development of immunotherapies capable of benefitting certain subgroups of melanoma patients.^[5]^

Viscum album L. grows as a hemi-parasitic shrub (European mistletoe) on different host trees and contains a variety of biologically active compounds, particularly mistletoe lectins (ML) and viscotoxins (VT).^[6]^ Viscum album extracts (VAEs) and their compounds show antineoplastic activities, including cytotoxic and apoptogenic effects, immune stimulation, downregulation of tumor genes, inhibition of tumor cell migration, and neo-angiogenesis.^[6–10]^ Various standardized commercial VAEs are available in injectable preparations (usually used subcutaneously, but occasionally intravenously and intratumorally).^[11]^ Clinical trials found an improved quality of life in VAE-treated cancer patients and longer survival in patients with advanced pancreatic cancer.^[12,13]^ A randomized trial on high-risk MCM compared adjuvant VAE, interferon-alpha, or interferon-gamma treatment with an untreated control group and found no benefit for any intervention.^[14]^ A pharmaco-epidemiological cohort study on MCM (UICC/AJCC stage II/III) found a prolonged cancer-specific, overall, and disease-free survival and less metastases in lung and brain in the VAE-treated patients.^[15]^ Remissions of various tumors—including MCM—have been described in case reports and small trials.^[16–24]^ Frequent side effects of VAE treatment include self-limited, dose-dependent, inflammatory local skin reactions, and flu-like symptoms. Occasionally, pseudo-allergic reactions can occur. Regardless, VAE therapy is, for the most part, safe, even at higher doses.^[25]^

## 2 Case presentation

A 61-year-old Caucasian woman was diagnosed with a stage 1a MCM of the left auricle [pT1a, N0 (negative sentinel lymph node), cM0, vertical tumor thickness 1.0 mm]. The patient is a former teacher, currently working as a therapist. In terms of UV exposure, the patient reported frequent canoeing trips in the summer as a young woman, during which the head was covered with a cap that possibly did not protect the ears.

**Figure 1:**
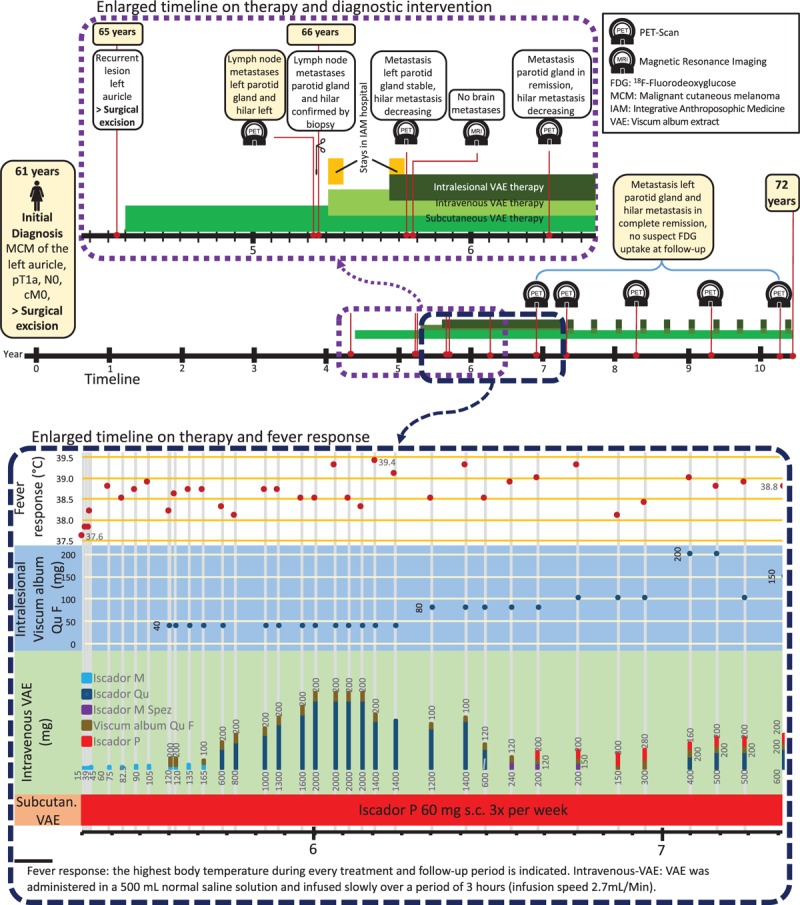
Timeline of the patient with enlarged illustrations of the 3 years after recurrence of the tumor and the 2 years of intensified VAE therapy.

**Figure 2:**
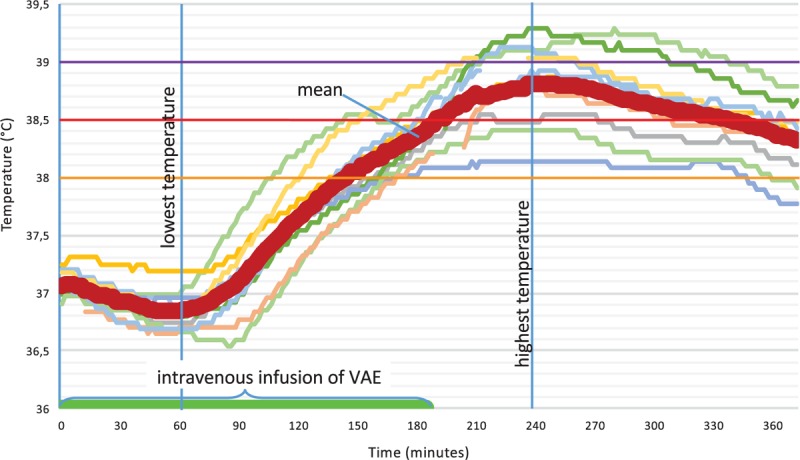
Course of body temperature during 10 sample treatments with intravenous infusions of VAE in the day clinic. Temperature was continuously recorded with a rectal/vaginal temperature probe.

The tumor was treated with surgical excision (BRAF mutation analysis was negative). After 4 years and 4 months, the MCM recurred at the left auricle and again was excised. In addition, the patient started a treatment with subcutaneous VAE injections [Iscador P c Hg Series I (vials of 0.1–10 mg) 3 times per week]. Eleven months later, a positron emission tomography–computed tomography (PET-CT) showed lymph node metastases in the region of the left parotid gland (8 × 7 mm) and hilar (26 × 19 mm). These results were confirmed by fine needle puncture biopsy. Systemic therapy (Dacarbazine/Ipilimumab) was recommended, but the patient decided against it. Instead, over the next 2 years, VAE therapy was intensified in dosage, preparation, and application form to yield more aggressive treatment of the tumor tissue:

1) Subcutaneous injections were changed to Iscador P 60 mg 3 times weekly (for detailed information on the VAE preparations used, see Table 1).^[26,27]^
2) Intravenous infusions—about every 2 weeks—were applied with increasing VAE dosage to provoke fever reactions to stimulate the immune system against the tumor tissue; dosage of VAE was adjusted after these reactions. In total, the patient received 36 intravenous VAE treatments during 2 years with doses up to 2200 mg VAE (containing 171,600 ng/ML and 6700 μg VT). VAE-induced temperature reactions and fever are described in Fig. 1 and displayed in a characteristic time curve in Fig. 2.
3) Intralesional application was performed into the parotid gland metastasis (Viscum album Qu F, compounded preparation, unfermented, Lot 3186/3033/4138/3138) in increasing dosage up to 200 mg VAE (containing 58,100 ng/ML and 585 μg VT) to yield a cytotoxic reaction against tumor cells, increased immune processing of tumor particles, and an increased immune response against tumor tissue. During 2 years, the patient received 26 intralesional VAE applications (Fig. 1).

**Table 1:**
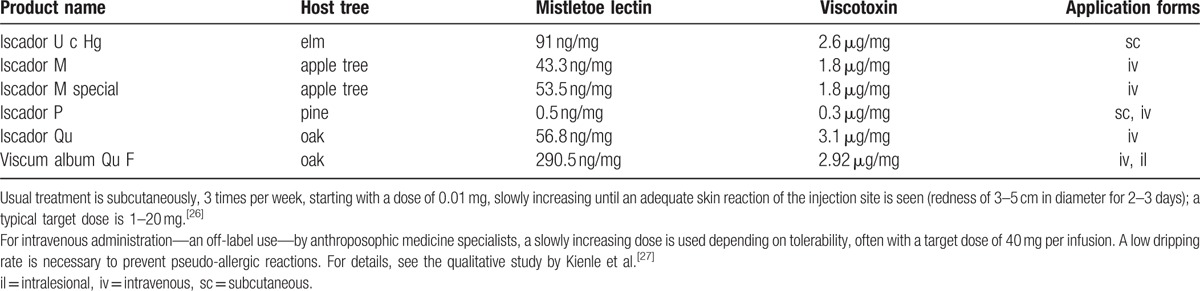
Viscum album extract (VAE) preparations.

After the intensified treatment period of 2 years, subcutaneous VAE treatment was continued until the present day, and the other application types were continued at longer intervals. Side effects included fever-associated symptoms such as body aches, shivering, and sometimes strong nausea, which were restricted to the treatment and fever period; skin reactions at the injection sites were self-limiting within 2 to 3 days. The performing physician had extensive experience with high-dose VAE therapy as well as training in intensive care and emergency medicine. Apart from the described symptoms, the treatment was well tolerated and no other side effects occurred during the whole course.

**Figure 3:**
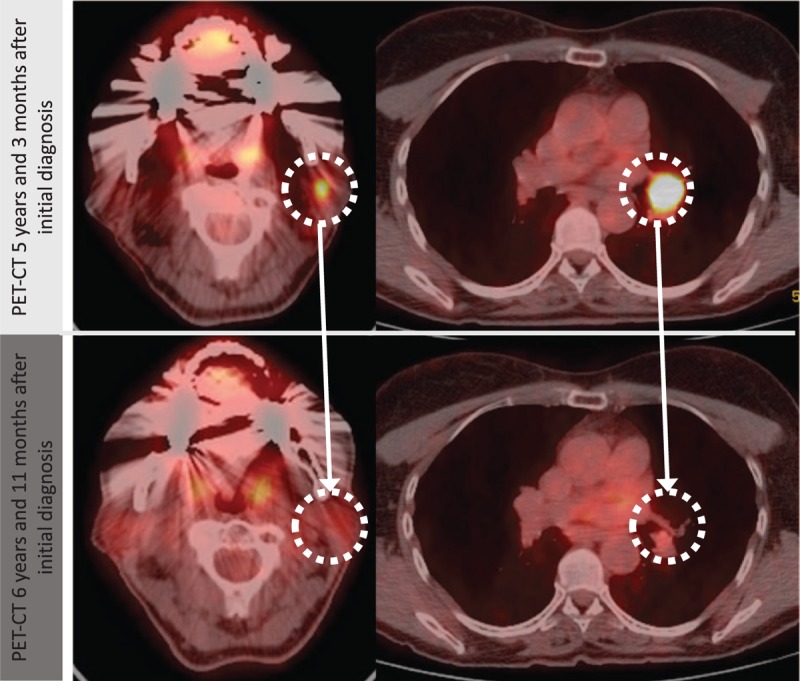
PET-CT of the first diagnose of a regional (cervical) and hilar lymph node metastases and the follow-up scan where the metastases are in complete remission.

The follow-up PET-CT examinations showed regressing local and hilar metastases. Twelve months after the initiation of intensified intravenous and intralesional VAE treatment, no further fludeoxyglucose (^18^F) (FDG)-uptake was seen in the left parotid gland metastasis, and the hilar lesions had significantly decreased. After a further 12 months, no suspect lesions could be detected in PET-CT (Fig. 3). Follow-up examinations, conducted every 12 months, showed no signs of malignancy. The patient experienced no severe adverse events; regular complete blood counts (erythrocytes, thrombocytes, leukocyte differential count) showed no deviation from the normal range; natural killer cells, however, were low before initiation of VAE therapy (78 cells/μL) and stayed low throughout the intervention (range of 73–137 cells/μL).

During the whole period of treating the metastatic disease, no other tumor-specific therapy was applied. At the time of this publication, 5 years after diagnosing metastatic disease (10.5 years after the initial diagnosis), the patient is tumor free, is in good health, and has no functional limitations.

## 3 Antecedent and concomitant therapies

In this case, high-dose VAE therapy was used within an individualized therapy concept of integrative anthroposophic medicine.^[28]^ No other cancer-specific medical treatments were used.

The patient also suffered from advanced osteochondrosis intervertebralis, goiter, and migraine. Fourteen years before the initial diagnosis of MCM, the patient had been diagnosed with carcinoma in situ of the cervix, which was treated with laser conization. Forty-three years before diagnosis with MCM, she suffered a cranial base fracture.

The patient participated in seminars on mental healing, provided by her health insurance, where she worked on negative life events from her early childhood.^[29]^ In addition to nutritional supplements containing sodium, calcium, potassium, and magnesium, the patient took fish oil capsules.

## 4 Patient's view

Following the diagnosis of the metastases, I was told at the University Hospital that it would most likely be a month before further therapy was continued. During this time, I found out about intensive Mistletoe Therapy and the hospital offering Anthroposophic Medicine. I learnt that I could have confidence in myself and that I could contribute to my own self-healing process. I realized that my own commitment to becoming healthy was needed, and learned to be grateful for everything that was healthy in me. This commitment to my own health also involved the development of an awareness of my life, including uncovering all those things which were sabotaging me from the inside. Because of this, I was able to find and take my own path in therapy—even if there were doctors who wanted to convince me not to take this path. I was able to tolerate the Mistletoe Therapy, with its high fever and side effects, as well as the sometimes painful injections, because I felt safe and confident in the treatment.

## 5 Discussion

We present a case of metastasizing MCM treated with VAE. Initial doses were low as per usual. Following metastatic diagnosis, however, doses were gradually intensified to very high levels with fever induction, and combinations of application forms (subcutaneous, intravenous, intralesional) were used. No other tumor-specific treatment was used. This treatment resulted in complete remission of all tumor lesions and long-term survival characterized by general good health and freedom from physical limitations.

Other case reports of VAE in MCM have also reported complete, long-lasting remission, and long-term survival.^[16,17,22]^ Von Laue^[16]^ described the interesting observation that injections of VAE into a subcutaneous metastasis of MCM led to its regression and also to the regression of a brain metastasis. Following subcutaneous low-dose VAE treatment, however, new metastases developed, but these again regressed under high-dose VAE treatment.^[16]^ VAE is cytotoxic and stimulates a variety of immune responses (e.g., proliferation and antigen presentation of dendritic cells, proliferation of CD4^+^ T cells, increase of natural killer cell-mediated cytotoxicity, release of cytokines including the pyrogens interleukin 1 and 6).^[10]^ The phenomenon observed by von Laue^[16]^ may therefore be explained by an increased release of tumor-associated antigens after cytotoxic action of VAE, as well as enhanced immunologic recognition and increased immune response against the tumor tissue—all triggered by VAE treatment. The same mode of action may be assumed in our case, where intralesional treatment of the parotid gland metastasis led to complete remission of all cancer lesions, including the treated metastasis.

ML is structurally related to shiga-toxin, acts as a pattern recognition receptor ligand, and thereby activates dendritic cells needed for a full-blown T-cell response against cancer cells.^[30]^ This mode of action may explain the highly inflammatory responses with elevated body temperature that occur after intravenous high-dose VAE treatment. This clinical picture also resembles tumor remissions after administration of fever-inducing bacterial vaccines (*S pyogenes* and *Serratia marcescens—“*Coley's toxins*”*) or the spontaneous remissions observed in the course of Erysipelas.^[31–33]^ Fever-inducing high-dose VAE treatments with subsequent relevant, long-lasting tumor remissions have been described in trials on hepatic and breast cancer as well as in case reports and case series concerning various cancers including MCM.^[16,18,19,22,23,34,35]^

From these preclinical and clinical observations, we presume that the immunological properties of VAE, especially those of its lectins, play a key role in the tumor response to VAE treatment in this case.

## 6 Safety aspects

This case describes an exceptional high-dose VAE treatment (even compared with other high-dose VAE treatments in case reports and studies) without any severe or persisting side effects. The treatment was carried out by a physician with extensive experience in high-dose VAE treatment. Before treatment and during the whole treatment period, the patient was healthy and in good condition and without comorbidities. Regular blood counts of the patient were normal throughout the intervention and showed no indication for hematoxicity or immunotoxicity of these very high VAE doses. It has to be considered, however, that we report the results of only a singular case. The treatment strategy therefore cannot replace the recommended standard multimodal treatment. VAE treatment, while safe even in higher doses,^[25]^ still produces high fever reactions, which can be too strenuous for patients with advanced disease or those of reduced physical or mental health or undergoing contemporaneous chemo-/radiotherapy. In rare cases, intravenous VAE infusions—an off-label use of VAE—may induce allergic or pseudo-allergic reactions, especially when the administration rate is too fast. Patients should therefore give informed consent and remain under medical supervision, and doctors should be equipped to treat allergic or pseudo-allergic reactions.^[27,36]^

## 7 Conclusion

On the basis of this case and earlier reported cases, VAE seems to have a positive potential in MCM, especially when used in high dosages. ML and VT are known to have direct cytotoxic properties and stimulate immune pathways of the specific and unspecific immune system and in this way might have contributed to tumor control in this case. Further research on the safety and effectiveness of high-dose and intravenous and intralesional VAE, particularly in MCM, is warranted.
